# Systematic review automation technologies

**DOI:** 10.1186/2046-4053-3-74

**Published:** 2014-07-09

**Authors:** Guy Tsafnat, Paul Glasziou, Miew Keen Choong, Adam Dunn, Filippo Galgani, Enrico Coiera

**Affiliations:** 1 Centre for Health Informatics, Australian Institute of Health Innovation, University of New South Wales, Sydney, Australia; 2 Centre for Research on Evidence Based Practice, Bond University, Gold Coast, Australia

**Keywords:** Systematic reviews, Process automation, Information retrieval, Information extraction

## Abstract

Systematic reviews, a cornerstone of evidence-based medicine, are not produced quickly enough to support clinical practice. The cost of production, availability of the requisite expertise and timeliness are often quoted as major contributors for the delay. This detailed survey of the state of the art of information systems designed to support or automate individual tasks in the systematic review, and in particular systematic reviews of randomized controlled clinical trials, reveals trends that see the convergence of several parallel research projects.

We surveyed literature describing informatics systems that support or automate the processes of systematic review or each of the tasks of the systematic review. Several projects focus on automating, simplifying and/or streamlining specific tasks of the systematic review. Some tasks are already fully automated while others are still largely manual. In this review, we describe each task and the effect that its automation would have on the entire systematic review process, summarize the existing information system support for each task, and highlight where further research is needed for realizing automation for the task. Integration of the systems that automate systematic review tasks may lead to a revised systematic review workflow. We envisage the optimized workflow will lead to system in which each systematic review is described as a computer program that automatically retrieves relevant trials, appraises them, extracts and synthesizes data, evaluates the risk of bias, performs meta-analysis calculations, and produces a report in real time.

## Background

Evidence-based medicine stipulates that all relevant evidence be used to make clinical decisions regardless of the implied resource demands [1]. Systematic reviews were invented as a means to enable clinicians to use evidence-based medicine [2]. However, even the conduct and upkeep of updated systematic reviews required to answer a significant proportion of clinical questions, is beyond our means without automation [3-9].

Systematic reviews are conducted through a robust but slow and resource-intensive process. The result is that undertaking a systematic review may require a large amount of resources and can take years [10]. Proposed decision support systems for systematic reviewers include ones that help the basic tasks of systematic reviews [6,12,13]. The full automation of systematic reviews that will deliver the best evidence at the right time to the point of care is the logical next step [14]. Indeed, research into automatic systematic review systems is distinct from - and related to - research on systematic reviews [5].

**Figure 1:**
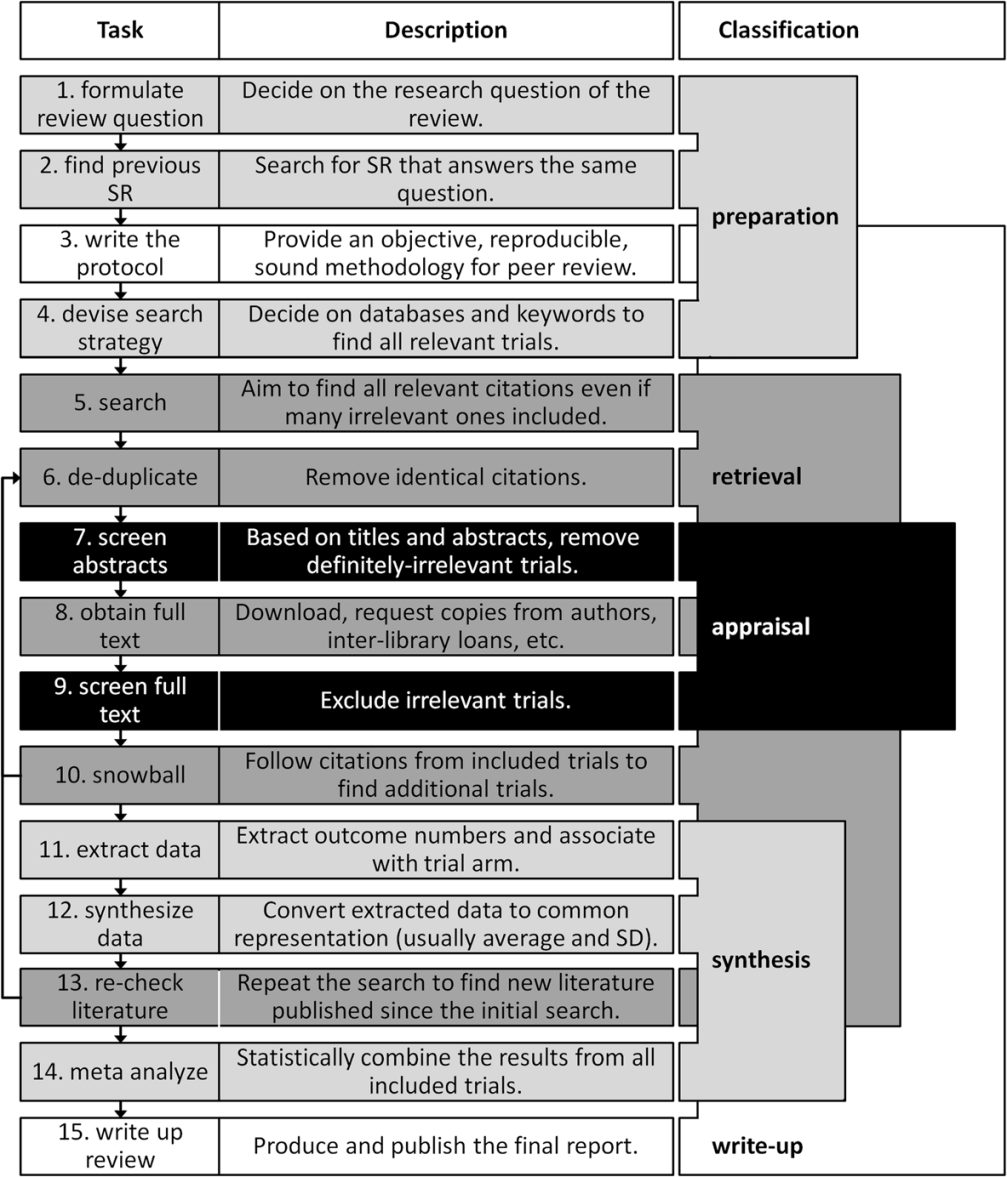
**Existing methods for systematic reviews follow these steps with some variations.** Not all systematic reviews follow all steps. This process typically takes between 12 and 24 months. Adapted from the Cochrane [10] and CREBP [11] Manuals for systematic reviews. *SR* systematic review, *SD* standard deviation.

The process of creating a systematic review can be broken down into between four and 15 tasks depending on resolution [5,10,13,15]. Figure 1 shows both high-level phases and high-resolution tasks encompassed by the phases. The process is not as linear and compartmentalized as is suggested by only the high-level view. In this article, we review each task, and its role in automatic systematic reviews. We have focused on systematic reviews of randomized controlled trials but most of the tasks will apply to other systematic reviews. Some tasks are not amenable to automation, or have not been examined for the potential to be automated; decision support tools for these tasks are considered instead. In the last section of this paper, we describe a systematic review development environment in which all manual tasks are performed as part of a definition stage of the review, and then a software agent automatically creates and checks the review according to these definitions when needed and at a push of a button.

## Discussion

### What should and should not be automated

The process of designing a systematic review is in part creative and part technical. We note that systematic review protocols have a natural dichotomy for tasks: creative tasks are done during the development of the question and protocol and technical tasks can be performed automatically and exactly according to the protocol. Thus, the development of the review question(s) is where creativity, experience, and judgment are to be used. Often, the protocol is peer-reviewed to ensure its objectivity and fulfillment of the review question [10]. Conducting the review should then be a matter of following the review protocol as accurately and objectively as possible.

In this scenario, the review protocol is developed much like a recipe that can then be executed by machine [5]. Tasks are reordered so that necessarily manual tasks are shifted to the start of the review, and automatic tasks follow. As an example, consider risk of bias assessment which sometimes requires judgment depending on the outcome measures, the intervention and the research question. During the review protocol preparation, a reviewer would train the system to make the required specific judgment heuristics for that systematic review. A classification engine will use these judgments later in the review to appraise papers. Updating a review becomes a matter of executing the review at the time that it is needed. This frees systematic reviewers to shift their focus from the tedious tasks which are automatable, to the creative tasks of developing the review protocol where human intuition, expertise, and common sense are needed and providing intelligent interpretations of the collected studies. In addition, the reviewers will monitor and assure the quality of the execution to ensure the overall standard of the review.

The automation of some tasks may seem impossible and fantastic. However, tools dedicated to the automation of evidence synthesis tasks serve as evidence that what seemed fantastic only a few decades ago is now a reality. The development of such tools is incremental and inevitably, limitations are, and will remain, part of the process. A few examples are given in Table 1.

**Table 1:** Examples of tools used for the automation of evidence synthesis tasks

**Step**	**Example application**	**Description**	**Limitations**
Search	Quick Clinical	Federated meta-search engine	Limited source databases not optimized for systematic reviews
Search	Sherlock	Search engine for trial registries	Limited to clinicaltrials.gov
Search	Metta	Federated meta-search engine for SR	Not available publicly
Snowballing	ParsCit	Reference string extraction from published papers	Does not fetch nor recursively pursue citations
Screen titles and abstracts	Abstrackr	Machine learning -based abstract screening tool	May reduce review recall by up to 5%
Extract data	ExaCT	PICO and other information element extraction from abstracts	No association (e.g. of outcome with trial arm), results only available in HTML
Extract data	WebPlotDigitizer	Re-digitization of data from graphs and plots.	No support for survival curves, no optical character recognition
Meta-analyze	Meta-analyst	Create a meta-analysis from extracted data	Limited integration with data-extraction and conversion programs
Write-up	RevMan-HAL	Automatic summary write-up from extracted data.	Only works with RevMan files
Write-up	PRISMA Flow Diagram Generator	Automatic generation of PRISMA diagrams	Does not support some complex diagrams

### Finding decision support tools for systematic reviewers

We have searched PubMed, Scopus, and Google Scholar for papers that describe informatics systems that support the process of systematic reviews, meta-analysis, other related evidence surveying process, automation of each of the tasks of the systematic review. We have used a broad definition of automation that includes software that streamline processes by automating even only the trivial parts of a task such as automatically populating database fields with manually extracted data. We excluded manually constructed databases even if an application programming interface is provided, although we did not necessarily exclude automatic tools that make use of such databases. We searched for each systematic review task names, synonymous terms as well as names of the algorithms used in papers on automation and systematic reviews. We tracked citations both forward and backward and included a representative set of papers that describe the state of the art.

### How individual tasks are supported

This section describes each of the systematic review tasks in Figure 1 in detail. Each task is described along with the potential benefits from its automation. State-of-the-art systems that automate or support the task are listed and the next stages of research are highlighted.

#### Task 1: formulate the review question

##### Task description

There is no single correct way to formulate review questions [16]; although, it is desirable to prioritize review questions by burden of disease for which there is a lack of review in the area. While choosing a review topic is not constrained, factors such as expertise in the area and personal interest are common [16]. All research questions need to be presented with sufficient detail so as to reduce ambiguity and help with the critical appraisal of trials that will be included or excluded from the review. The population, intervention, control, and outcome (PICO) were recommended as elements that should be included in any question [1,17]. A well-written review question provides logical and unambiguous criteria for inclusion or exclusion of trials.

##### Automation potential

Automatic systems can help identify missing evidence, support creative processes, and support the creative processes of identifying questions of personal interest and expertise. Prioritization of questions can save effort that would otherwise be spent on unimportant, irrelevant or uninteresting questions and duplication. Decision support for question formulation can ensure questions are fully and unambiguously specified before the review is started.

##### Current systems

Global evidence maps [18,19] and scoping studies [20] are both literature review methods designed to complement systematic review and identify research gaps. Both differ from systematic review in that they seek to address broader questions about particular areas rather than answer specific questions answered by a narrow set of clinical trials. Scoping studies are relatively quick reviews of the area. Global evidence maps are conducted in a formal process, similar to systematic reviews, and thus may take in excess of 2 years [21]. Broad reviews direct reviewers towards questions for which there is a gap in the evidence. Algorithms that can extract the evidence gaps identified in such reviews can provide decision support to systematic reviewers choosing a research question.

##### Future research

Research for additional decision support tools should focus on identification of new problems. Work in the field of artificial intelligence on hypothesis generation and finding [22-24] may provide an automatic means of suggesting novel review questions. Economic modeling tools and databases that can automatically assess burden of disease according to established standards [25] can help prioritize potential review questions.

Question prioritization might not be required when systematic reviews can be conducted quickly and economically. An exhaustive set of research questions can be asked about the suitability of any treatment for any population. While the number of combinations of questions and answers poses its own challenges, automatic synthesis will quickly determine that most condition-treatment pairs have no evidence (e.g. because they are nonsensical) and will not dedicate much time to them.

#### Task 2: find previous systematic reviews

##### Task description

The resources and time required to produce a systematic review are so great that independently creating a duplicate of an existing review is both wasteful and avoidable [26]. Therefore, if the reviewer can find a systematic review that answers the same question, the potential savings might be substantial. However, finding a previous systematic review is not trivial and could be improved.

##### Automation potential

An accurate and automatic system that finds previous systematic reviews given a clinical question will directly reduce the effort needed to establish whether a previous review exists. Indirectly, such a system can also reduce the number of redundant reviews. Even if an out-of-date systematic review is identified, updating it according to an established protocol is preferable to conducting a new review.

##### Current systems

Databases of systematic reviews include the Cochrane Database of Systematic Reviews [6], Database of Abstracts of Reviews of Effects (DARE), the International Register of Prospective Systematic Review (PROSPERO) [27], SysBank [28], and PubMed Health. These should be searched before a new review is started.

Search strategies and filters have been suggested to improve recall (the proportion of relevant documents retrieved out of all relevant documents) and precision (the proportion of relevant documents retrieved out of all retrieved documents) respectively of search queries [29-34]. Specific search filters designed to find systematic reviews have been proposed [29,35]. PubMed's Clinical Queries feature adds a filter [32] that restricts searches to clinical trials and systematic reviews. PubMed Health provides a search filter that works well for finding systematic reviews but is limited to PubMed and DARE currently [36].

Automation of this task is to find systematic reviews that answer the research question without manually translating research questions into search queries. Question answering systems have been in development since the 1960s [37]. For this task, a form of an automatic web-based question-answering system [38,39] in which the answer to the review question is a systematic review, is most suitable.

##### Future research

Research is needed on the design and evaluation of specialized question-answering systems that accept the nuances of systematic review questions, use search filters and strategies to search multiple databases to find the relevant systematic review or systematic review protocol.

#### Task 3: write the protocol

##### Task description

Writing the systematic review protocol is the first step in planning the review once it is established that the review is needed and does not already exist. This task requires specialized expertise in medicine, library science, clinical standards and statistics. This task also requires creativity and close familiarity with the literature in the topic because reviewers must imagine the outcomes in order to design the questions and aims. As there are currently no methods to formally review the consistency of the protocol, peer-review is used to ensure that the proposed protocol is complete, has the potential to answer the review question, and is unbiased.

##### Automation potential

Writing the review protocol formally will allow it to be automatically checked for consistency and logical integrity. This would ensure that the protocol is consistent, unbiased, and appropriate for the research question. Verified protocols can thus reduce or remove the need for protocol peer review altogether.

##### Current systems

Systems that are used to support the writing of systematic review protocols include templates and macros. Cochrane's Review Manager [40] uses a protocol template: it has standard fields that remind the reviewer to cover all aspects of a Cochrane protocol, including inclusion criteria, search methods, appraisal, extraction, and statistical analysis plan. Improvement of the templates is subject to ongoing research [40,41].

##### Future research

Automation and support for protocol writing can include reasoning logic that checks the completeness of the inclusion criteria (e.g., by checking that the population, intervention, and outcome are all specified), warns of potential biases in the inclusion criteria (e.g., that the disease being researched is more prevalent in different age groups but the protocol does not account for age), and tests the consistency and feasibility of the inclusion criteria (e.g., warns that a population of women with prostate cancer is probably wrong).

This sort of consistency verification is a type of computational reasoning task that requires models of the problem to be created through knowledge representation, simulation, and/or constraint resolution [42]. Such models for clinical trials were proposed in the 1980s [43], are still evolving [44], and similar models should also be possible for other clinical questions.

Language bias remains an unanswered problem with little existing systems available to address it [45]. Automatic information systems present an opportunity to mitigate such bias. NLP algorithms for non-English languages, including optical character recognition (OCR) of languages not using a Latin alphabet, are required for such systems.

#### Task 4: devise the search strategy

##### Task description

Devising the search is distinct from conducting the search. The correct search strategy is critical to ensure that the review is not biased by the easily accessible studies. The search strategy describes what keywords will be used in searches, which databases will be searched [10], if and how citations will be tracked [46,47], and what and how evidence will be identified in non-database sources (such as reference checking and expert contacts) [48]. All Cochrane systematic review protocols, and many others, undergo peer review before the search is conducted.

##### Automation potential

Automatic search strategy creation by using the PICO of the planned question could form part of a decision support tool that ensures the strategy is consistent with the review question, includes both general and specific databases as needed, and that tested citation tracking methodologies are used.

##### Future research

Decision support tools for search strategy derivation could also suggest data sources, keywords (e.g., MeSH terms [49]) and search strategies [30,34,50] most suitable for the research question. It could also warn against search strategies that are too tailored to a particular database and that would thus be ineffective in others. This may involve understanding of the indexing terms (e.g., MeSH versus EMTREE) and structures of different databases (e.g., PubMed versus EMBASE).

Research into natural language processing is needed for algorithms that can understand a clinical question in a rich sense: extract its context, classify it into a type of clinical question, and/or identify pertinent keywords from the research question. An up-to-date database of clinical papers classified by type is needed to help decision support systems find, identify, and suggest the most suitable databases for the task.

#### Task 5: search

##### Task description

The biomedical literature is the main source of evidence for systematic reviews; however, some research studies can only be found outside of literature databases - the so called grey literature - and sometimes not at all [51,52]. For reviews to be systematic, the search task has to ensure all relevant literature is retrieved (perfect recall), even at the cost of retrieving up to tens of thousands of irrelevant documents [52] (precision as low as 0.3% [52]). It also implies that multiple databases have to be searched. Therefore, reviewers require specific knowledge of dozens of literary and non-literary databases, each with its own search engine, metadata, and vocabulary [53-55]. Interoperability among databases is rare [56]. Variation among search engines is large. Some have specialized query languages that may include logical operators such as OR, AND, and NOT, syntax for querying specific fields such as ‘authors’ and ‘year’, operators such as ADJ (adjacent to) and NEAR, and controlled keyword vocabularies such as Medical Subject Heading (MeSH) [49] for PubMed, and EMTREE for EMBASE.

Studies that investigated the efficacy of trial registration found that only about half of the clinical trials are published [57] and not all published trials are registered [58]. This indicates that registries and the literature should both be searched.

The most commonly used databases in systematic reviews are the general databases MEDLINE, Cochrane Library, and EMBASE [55] but hundreds of specialty databases also exists, e.g., CINAHL for nursing. The main non-literary sources are the American clinicaltrials.gov, the World Health Organization's International Clinical Trials Registration Platform [59], and the European clinicaltrialsregister.eu.

Studies that measured overlap between PubMed (which covers MEDLINE) and EMBASE found only a small number of articles that appeared in both [60-62]. These studies show why searching multiple databases is important. A study that measured overlap between Google Scholar and PubMed found Google Scholar to lead to the retrieval of twice as many relevant articles as well as twice as many irrelevant ones [63]. This study indicates that simply merging all databases into one large database improves recall without requiring searching multiple databases, but does not accelerate the systematic review.

##### Automation potential

Automation of the search task will reduce the time it takes to conduct the search and ensure that the translation of the generic search strategy into database-specific queries retains the integrity of the protocol. More comprehensive search (i.e., increased recall) will see to the inclusion of more evidence in the review. Better targeted search (i.e., increased precision) can reduce the number of papers that need critical appraisal in later stages.

##### Current systems

Decisions support for searching can utilize algorithms of at least one of the following: (i) algorithms that increase the utility (in terms of precision and recall) of user-entered queries and (ii) algorithms that help users write better search queries. Some web search engines already use a combination of both classes (e.g., Google).

Searching for clinical trials can be supported by specifically crafted filters and strategies [29-34]. Automatic Query Expansion (AQE) is the collective name for algorithms that modify the user's query before it is processed by the search engine [64]. Typically, AQE algorithms are implemented in the search engine itself but can also be provided by third parties. For example, HubMed (http://www.HubMed.org) provides alternative AQE algorithms unrelated to those offered by PubMed and without association with the National Library of Medicine which hosts MEDLINE. Examples of AQE algorithms include the following:

• Synonym expansion - automatically adding synonymous keywords to the search query to ensure that papers that use the synonym, and not the keyword used by the user, are retrieved in the search results;

• Word sense disambiguation - understanding keywords in the context of the search, replacing them with more suitable synonyms and dictionary definitions and removing unhelpful keywords; and

• Correct spelling mistakes and replace contractions, abbreviations, etc., with expanded terms more likely to be used in published work.

Document clustering algorithms group similar documents in a database. Search results are then computed by the database as document clusters that partially match the search query (i.e., only some of the clusters' members match the query) [65]. Clustering algorithms differ in the manner in which they identify documents as similar (i.e., by their similarity functions). Examples of document similarity measures include ones based on the terms that appear in the documents [66], and ones based on papers they cite [67].

A limited number of search engines help the user come up with better search queries. Google, and now many other web search engines, use keyword suggestion as soon as the user begins to type a search query, usually by suggesting terms that other users suggested previously. It may be more appropriate in the medical domain, to use a medical dictionary rather than previous searches, for example, to reduce spelling mistakes.

Meta-search engines are systems that amalgamate searches from several source databases. An automatic systematic review system that queries multiple databases would thus be a kind of meta-search engine by definition. An example of a basic type of meta-search engine is QUOSA (Quosa Inc. 2012). It lets the user query several source databases individually, and collects the results into a single list.

Federated search engines are meta-search engines that allow one to query multiple databases in parallel with a single query. Federated search engines need to translate the reviewer's query into the source databases' specific query languages and combine the results [68-70].

**Figure 2:**
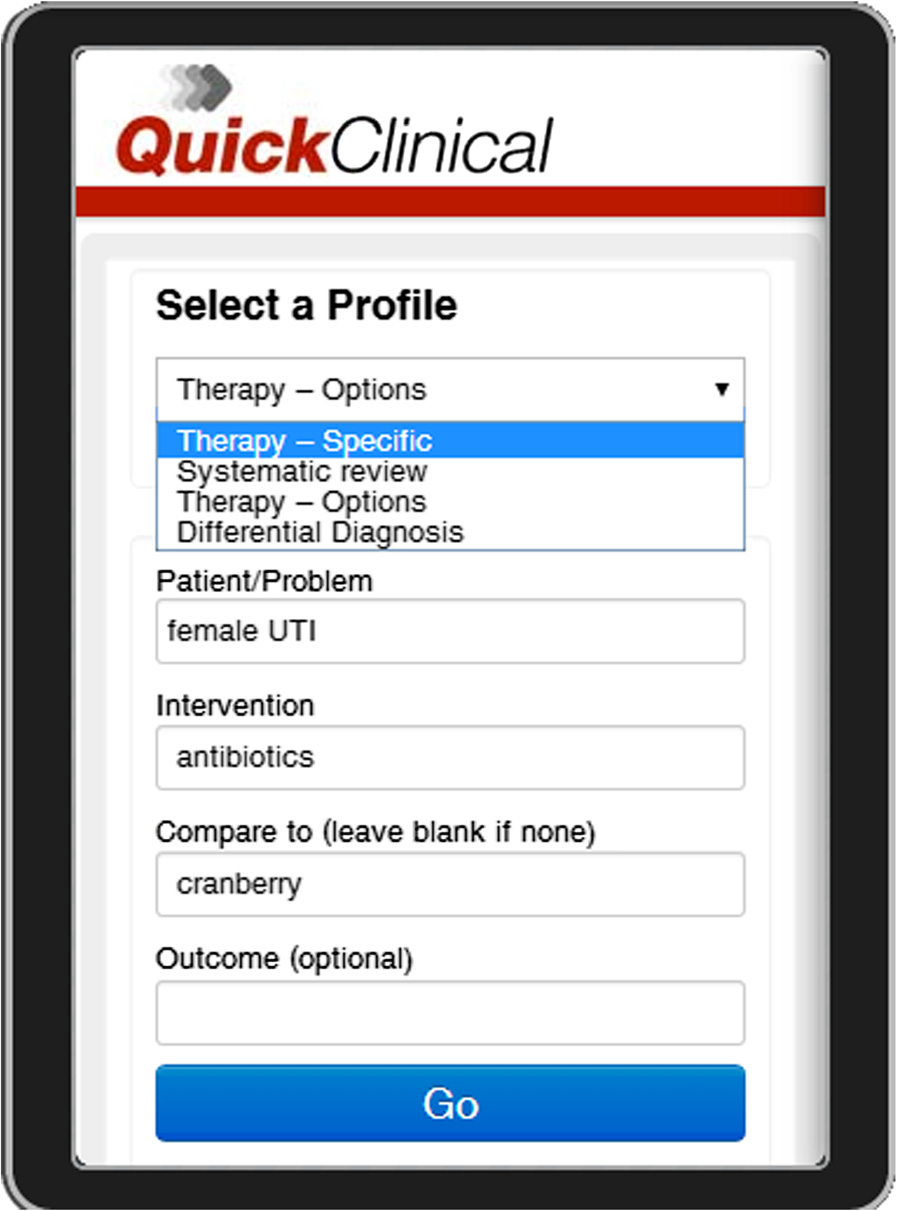
**A screen capture of the Quick Clinical query screen from the smartphone app version.** The Profile pull-down menu lets one select the class of question being asked (e.g. medication, diagnosis, patient education). The query fields are chosen to suit the question class. The four query fields shown (Disease, Drug, Symptom and Other) are taken from the Therapy question class.

Quick Clinical [70] is a federated search engine with a specialized user interface for querying clinical evidence. The interface prompts the user for a query class, and then for specific fields suitable for the chosen query class (Figure 2). This interface not only prompts users to elaborate on their questions, it also guides inexpert users on how to ask clinical questions that will provide them with meaningful results [71]. Quick Clinical's AQE algorithm enriches keywords differently depending on which field they were typed in [70].

The Turning Research Into Practice (TRIP) [72] database uses multiple query fields including to search PubMed. The query fields for population, intervention, comparison, and outcome (PICO) to enrich the query with terms that improve precision. In this way, TRIP uses knowledge about evidence-based medicine to improve evidence searches. TRIP is designed to provide quick access to evidence in lieu of a systematic review and its usefulness to systematic reviewers is yet to be demonstrated.

##### Future research

Expert searchers use iterative refinement of their search queries by a quick examination of the search results. As opposed to federated search engines which, in parallel, query each database once, expert searchers use a strategy of a series of queries to one database at a time. The automation of such a sequential searching is not yet explored.

The automatic translation of search queries between literature databases is not trivial. While general technical approaches exist and can convert the standard Boolean operators [70], these have yet to be demonstrated on translations that include database-specific vocabularies such as MeSH and EMTREE. BioPortal [73] is an ontology that maps vocabularies and includes MeSH but still lacks mappings to EMTREE or other literary database vocabularies.

A recent push to register all trials [74] has led to the creation of trial registries by governments [75-77] and health organizations [78,79]. The feasibility for future evidence search engines making use of such registries to find linked trial results and/or contact information for trial administrators, should yet be investigated.

#### Task 6: de-duplicate

##### Task description

Whenever citations are obtained from multiple sources, combining the results requires removing duplicate citations from the result lists [80]. Challenges in citation de-duplication arise due to variation in indexed metadata (e.g., DOI, ISBN, and page numbers are not always included), misspelling (e.g., in article title, journal name), abbreviation formats and legitimately duplicate information (e.g., identical author lists, different authors with the same name).

If the same study has more than one report - possibly with different author lists, different titles, and in different journals - both papers should often be cited, but they should only be included in meta-analysis as one trial [81]. Citation information alone cannot resolve this type of duplication which is also called ‘studification’. Characteristics needed to detect duplicated trials are only present in the text of the articles. This type of de-duplication is called study de-duplications as it aims to identify two distinct reports of the same study.

##### Automation potential

Both types of de-duplication are largely manual and time-consuming tasks and their automation has the potential to save many days that clinical librarians routinely spent on them. Study de-duplication, although the rarer of the two, is only usually detected after data have been extracted from both papers, after the authors have been contacted or sometimes not at all. Automatic de-duplication of the study can thus save precious time and free reviewers and clinical librarians to pursue other tasks.

##### Current systems

Current technologies focus on citation de-duplication [80]. Many citation managers (e.g., EndNote [82], ProCite [83]) already have automatic search for duplicate records to semi-automate citation de-duplication quite accurately [78,79]. Heuristic [84], machine learning (ML) algorithms and probabilistic string matching are used to compare author names, journal, conference or book names (known as venue) and titles of citations. This task can thus depend on our ability to accurately extract field information from citation strings which is also subject to ongoing research [85-89].

##### Future research

Future research should focus on study de-duplication which is distinct from citation de-duplication. Study de-duplication is trivial after information is extracted from the trial paper; however, the aim is to prevent unnecessary information extraction. Study de-duplication could be the result of studies being presented in different ways, in different formats or at different stages of the study and therefore requires sophisticated NLP algorithms that can infer a relationship between such papers. Many plagiarism detection algorithms have mechanisms to understand paper structure and find similarities between papers [90-92]. Such algorithms may guide development of algorithms that extract relationships between trial papers. Trial registries with multiple associated papers, and databases of extracted trial data could be used for automatic study de-duplication. Databases of manually extracted trial data are already maintained by some Cochrane group editors for specific domains but are not yet available for exploitation by automatic systems.

#### Task 7: screen abstracts

##### Task description

As the main aim of the retrieval task is to retrieve all relevant literature (perfect recall), the aim of the appraisal tasks is to exclude all irrelevant literature inadvertently retrieved with it. When appraising scientific papers' relevance to a systematic review, the vast majority of documents are excluded [10]. Exclusion is based on the relevance of the evidence to the research question and often an evaluation of the risk of bias in the trial.

Appraisal is normally conducted in two phases. First, titles and abstracts are used to quickly screen citations to exclude the thousands of papers that do not report trials or are trials not in the target population, intervention or do not measure the right outcome. This saves the reviewers from the lengthy process of retrieving the full text of many irrelevant papers.

##### Automation potential

Despite the apparent brevity of the screening of title and abstract, due to the large number to be screened, this is an error-prone and time-consuming task. Decision support systems can highlight information for reviewers to simplify the cognitive requirements of the task and save time.

A second reviewer is usually needed to screen the same abstracts. The two reviewers may meet to resolve any disagreements. Automatic screening systems could thus be used as a tie-breaker to resolve disagreements, and/or replace one or both screeners.

##### Current systems

Decision support systems use NLP to automatically highlight sentences and phrases that reviewers are likely to use for appraisal [93,94]. Often, information highlighting algorithms focus on PICO elements reported in the trial [95-97] but may also focus on the size of the trial [97,98], and its randomization procedure [95,97].

Existing commercial decision support systems are focused on helping reviewers manage inclusion and exclusion decisions, flag disagreements between reviewers and resolve them sometimes automatically [99-101].

Automation approaches are to use ML to infer exclusion and inclusion rules by observing a human screener [102-106], a class of algorithms called supervised ML. The Abstrackr system [103,104] observes inclusion and exclusion decisions made by a human reviewer. The system then extracts keywords from the abstracts and titles of the screened documents and builds models that mimic the user's decisions. When enough included as well as excluded documents are observed by the system, Abstrackr can automatically continue to screen the remaining reports and thus halve the total workload that the human reviewer would otherwise need to bear.

Current appraisal systems can reduce the number of documents that need to be appraised manually by between 30% and 50%, usually at the cost of up to 5% reduction in recall [102-106]. Another demonstration of an automatic screening algorithm alerts about new evidence that should trigger a systematic review update [106]. That system, however, did not achieve reliably high precision and recall.

##### Future research

A simple approach is to use the document type associated with each document in PubMed to remove all documents not classified as clinical trials. However, this approach is not reliable because it only works for PubMed and document labeled as randomized controlled trials are not necessarily reports of trial outcomes but could also be trial protocols, reviews of clinical trials, etc. A more accurate approach is therefore to use document classes together with specific keywords [107]. An alternative approach, not yet tested in this domain, is to get experts to develop rule-based systems using knowledge-acquisition systems.

Heuristic and ML systems can be complementary, and combinations of these approaches have proven accurate in other domains [108]. In future automated systematic reviews, we are likely to see a combination of rule-based and machine-learning algorithms.

#### Task 8: obtain full text articles

##### Task description

Obtaining the full text of published articles is technical, tedious and resource demanding. In the current process (Figure 1), full-text fetching is minimized by breaking the screening task into two parts. Known obstacles to full text fetching are a lack of standardized access to literature, burdensome subscription models and limited archival and electronic access. Full text retrieval requires a navigation through a complex network of links that spans multiple websites, paper-based content and even email. The problems are more pronounced for older studies.

##### Automation potential

Automation of full text retrieval has the potential to drastically change the systematic review process. Some tasks, such as screening from abstracts, would become redundant as screening of full text is superior. Other tasks, such as de-duplication, would be simplified as there would not be a need to rely on meta-data. Citation tracking (see below) may lead to systems that retrieve the required evidence primarily through means other than search.

##### Current systems

OvidSP [109] and PubMed Central [110] provide unified access to journal articles from multiple databases and journals and thus support full text retrieval. Some databases such as Google Scholar [111] and Microsoft Academic Search [112] provide multiple links for most documents that include the primary publisher's link, institutional copies and links to other databases, which may offer additional access options. Some reference managers such as EndNote [82] and ProCite [83] have full text retrieval functions.

##### Future research

Research on intelligent software agents that can autonomously navigate from search results to full text articles along several possible routes is still needed. Such systems could also extract corresponding author name, if it is known, send e-mail requests, and extract attachments from replies. Trial registries already contain author information and may contain links to full text papers could be used for this purpose.

#### Task 9: screen the full text articles

##### Task description

In the second stage of appraisal, reviewers screen using full text describing the trials not previously excluded. In this phase, a more detailed look at the trial allows the reviewer to exclude or include trials by inspecting subtle details not found in the abstract. This task is different from screening using abstracts in that a more careful understanding of the trial is needed. Pertinent information may be reported in sentences that have to be read in context.

##### Automation potential

Despite the smaller number of trials to screen, each trial requires more time and attention. A second reviewer is often used, and agreement by consensus is sought which make the process even more resource and time demanding. Computational systems could automatically resolve disagreements or replace one or both reviewers to reduce resource demand.

##### Current systems

Decision support systems that screen abstracts may work on full-text screening unmodified. Elements that are not normally present in the abstract, such as figures and tables, and that contain information pertinent to appraisal, are not yet mined by current systems. Systems that do make use of such information have been proposed [94,113] but have not yet seen adoption in this domain.

##### Future research

Research is required to evaluate such systems for their reliability, and integrate them into systematic review support systems.

#### Task 10: ‘snowballing’ by forward and backward citation search

##### Task description

Limitations of keyword searching have led to the development of secondary analysis of search results [114]. One such analysis method is called snowballing [46], which recursively pursues relevant references cited in retrieved papers (also called citation tracking) and adding them to the search results. While this is a time-consuming step, it has been shown to improve retrieval [115], and is a recommended practice [46]. Unlike keywords search, snowballing does not require specific search terms but allows the accumulation of multiple searches from different publishing authors [47].

##### Automation potential

Automatic snowballing has the potential to increase recall by following more citations than a human reviewer would. A case study on forward citation tracking on depression and coronary heart disease has shown to identify more eligible articles and reduce bias [47]. A review on checking reference lists to find additional studies for systematic reviews also found that citation tracking identify additional studies [115].

When combined with automatic appraisal and document fetching, automatic snowballing can have a compound effect by iteratively following select citations until no new relevant citations are found. A recent study [116] examined the potential for using citation networks for document retrieval. That study shows that in most reviews tested, forward and backward citation tracking will lead to all relevant trials manually identified by systematic reviewers. Snowballing would benefit almost all searches (>95%) but does not completely replace database searches based on search terms.

##### Current systems

Automatic citation extraction tools [85-89] are core to snowballing systems. Some databases such as Web of Science and Microsoft Academic Search provide citation networks (for a fee) that can be traversed to find related literature. Those networks are limited to papers indexed in the same database.

##### Future research

The risk of an automatic snowballing system is that it can get out of control, retrieving more articles than it is feasible to appraise manually. Formal definitions of robust stopping criteria are therefore pivotal research in this area. Integration with automatic appraisal and fetching systems will help to improve the practical application of snowballing systems.

#### Task 11: extract data

##### Task description

Data extraction is the identification of trial features, methods, and outcomes in the text of relevant trials. In many trial papers, primary information is only published in graphical form and primary data needs to be extracted from these plots as accurately as resolution permits. Usually two reviewers perform the task independently and resolve disagreements by consensus. Extracting data from text is one of the most time-consuming tasks of the systematic review.

##### Automation potential

A great deal of expertise, time, and resources can be saved through partial and complete automation of this time-consuming task. As two reviewers perform it in parallel, a computer extractor could serve as an arbiter on disagreements, or replace one or both reviewers. The identification, extraction of the number of patients in each arm, and extraction and association of outcome quantities with the same arms, can be challenging even to experienced reviewers. The information may be present in several sections of the trial paper making the extraction a complex cognitive task. Even partial automation may reduce the expertise required to complete this task, reduce errors, and save time.

##### Current systems

The approach currently used to automate extraction of information from clinical trial papers has two stages. The first sub-task is to reduce the amount of text to be processed using information-highlighting algorithms [95-97]. The second sub-task is to associate extracted elements with experimental arms and with outcomes [117-119].

ExaCT is an information-highlighting algorithm [95]. It classifies sentences and sometimes phrases that contain about 20 elements used in information extraction and screening. The classes include PICO, randomization, and subclasses of elements. For example, subclasses of intervention elements include route of treatment, frequency of treatment, and duration of treatment. ExaCT can often distinguish between sentences that describe the intervention and from those that describe the control but does not associate, e.g., measured outcomes with each arm.

To identify intervention sentences, a highlighting algorithm may be used to identify drug names in the title of the document. A template [118] or a statistical model [119] is then used to associate the number of patients in a particular arm in the study. Each outcome in each arm can then be summarized by two numbers: number of outcome events in the study arm and number of patients in the same study arm. Extracting all outcomes for all arms of the trial then gives a structured summary of the trial.

Software tools for digitizing graphs use line recognition algorithms to help a reviewer trace plots and extract raw data from them. Graph digitization software are not specific to systematic reviews and thus support the more common graph types such as X-Y and polar plots [120-122], but not survival curves, which are more common in clinical trials.

##### Future research

Current methods are still limited to processing one sentence at a time which severely limits the number of trials they can process. Future research systems will be required to create of summaries of trials based on information extracted from multiple sentences in different sections of the trial full text. For example, the trial may specify the number of patients randomized to each arm in one sentence (e.g., ‘*Five hundred and seventy-seven (377 Japanese, 200 Chinese) patients treated with antihypertensive therapy (73.5% [n = 424] received concomitant ACEI), were given either once-daily olmesartan (10-40 mg) (n = 288) or placebo (n = 289) over 3.2 ± 0.6 years (mean ± SD).’*) [123] and the number of events in each arm in another (‘*In the olmesartan group, 116 developed the primary outcome (41.1%) compared with 129 (45.4%) in the placebo group (HR 0.97, 95% CI 0.75, 1.24; p = 0.791)’*) [123]. In another example, the primary outcome is specified in the methods section (e.g., ‘duration of the first stage of labor’) [124] and only a reference to it can be found in the results section (e.g., ‘the first stage’) [124]. New NLP methods are required to be able to computationally reason about data from such texts.

Current methods all assume that trials can be modeled using a simple branching model in which patients are either assigned to one group or another or drop out [125]. While this model is suitable for a large proportion of published trials, other study designs have not yet been addressed.

As extraction methods develop, they will increasingly understand more study designs such as cross-over studies. Computationally reasoning over trials with a different number of arms and which are not controlled against placebo or ‘standard care’ would require significantly more elaborate computational reasoning [42].

Specific tools to extract survival curves will be beneficial to systematic review research. Research is also required to use optical character recognition (OCR) to digitize text (e.g., axes labels, data labels, plot legends) and combine the information with data extracted from the plot.

Databases of manually extracted trial data are already maintained by some Cochrane group editors for specific domains and collected in CRS [126]. Automatic extraction can populate such databases [43]. The feasibility of using trial databases in automatic systems is yet to be tested.

#### Task 12: convert and synthesize data

##### Task description

Synthesis may first require the conversion of numerical results to a common format, e.g., a common scale or a standardized mean difference, so that trials can be compared. Normally, comparison of continuous distributions is done according to the average and standard deviation (SD). Comparison of discrete measures uses relative risk (RR) and later converted to number needed to treat (NNT).

##### Automation potential

Conversion between statistical formats requires specific expertise in statistics. Automation of this task can thus reduce the requirement for specific training in statistics and to reduce the chance of the wrong conversion being used, e.g., confusing standard error and standard deviations.

##### Current systems

It is standard practice to use statistical packages for synthesis. However, much of the practice is still manual, time consuming, and error prone. We have identified only one study that attempted automatic extraction and synthesis of discrete distributions to NTT and RR formats [118].

##### Future research

The automatic synthesis system will have to automatically recognize the outcome format (e.g., as a confidence interval, average effect size, *p* value, etc.), select the statistical conversion, and translate the outcome to its average and standard deviation. These formulae may require extracted entities that may not be reported as part of the outcome, such as the number of patients randomized to that arm.

#### Task 13: re-check the literature

##### Task description

Due to the time lag between the initial search and the actual review (typically between 12 and 24 months), a secondary search may be conducted. The secondary search is designed to find trials indexed since the initial search. Often this task implies a repetition of many previous tasks such as appraisal, extraction, and synthesis making it potentially very expensive.

##### Automation potential

In an automatic review system, retrieval, appraisal, extraction, and synthesis would occur in quick succession and in a short amount of time. This would make this task altogether unnecessary. Consequentially, an automatic review process will have the compounded effect that will save much manual labor.

#### Task 14: meta-analyze

##### Task description

For a systematic review to be useful to a clinician, it needs to be presented in a clear, precise and informative manner [127,128]. Systematic reviews are often followed by a meta-analysis of the included trial results. The most common way to summarize a meta-analysis is using a forest plot [129] but other plots exist, such as Galbraith diagrams (also called radial diagrams) and L'Abbé plots. Other plots are used for data checking purposes, e.g., funnel plots [130] show whether publication bias is likely to affect the conclusion of the systematic review.

##### Automation potential

Transferring quantities between software packages is time consuming and error prone. Thus, integration of synthesis and graphing software has the potential to save time and reduce errors.

##### Current systems

Much of the meta-analysis is already automatic as software for combining, comparing, and reporting the meta-analysis graphically, are already in wide use [131-134].

##### Future research

Plotting is routinely manual with the aid of specialized software. Software for systematic reviews are included in specific software packages including Review Manager (RevMan) [40], Meta-Analyst [131], MetaDiSc [132], and MetaWin [133], all of which already produce Forest and other plots.

#### Task 15: write up the review

##### Task description

Write-up of a scientific paper according to reporting standards [135] is the major dissemination channel for systematic reviews. This process is prolonged and subject to review, editing, and publication delays [4,7]. It involves many menial sub-tasks that can be automated, such as typesetting figures and laying out the text. Other sub-tasks, such as interpreting the results and writing the conclusion, may require more manual interventions.

##### Automation potential

The automatic creation of a report from the protocol can save years in the production of each systematic review. Rather than the protocol and the written-up report requiring separate peer-review, only the protocol would be reviewed as the report will be produced directly from it. This will reduce the load on peer reviews and reduce delays further.

##### Current systems

The Cochrane Collaboration provides templates for the production of the systematic review protocol and text. These help reviewers ensure a complete and consistent final product. RevMan-HAL [136] is a program that provides templates and rules that generate the results and conclusion sections of Cochrane reviews from RevMan files that contain a meta-analysis. After the template is filled in, the reviewer may edit the final report.

A well-written report includes graphical and textual summaries. In addition to the forest plots and the other meta-analysis representations mentioned above, graphical summaries include tables and PRISMA flow diagrams. Microsoft Office Excel is often used to enter data into tables and copy them into the report. Tools exist for creating relevant diagrams including forest plots and PRISMA flow diagram [137].

##### Future research

Natural Language Generation (NLG) technology [138] can be used to write specific paragraphs within the review such as descriptions of the types of documents found, results of the appraisal and summaries of the findings. Disparity in data formats among tools mean that errors may be introduced when manually transferring data between tools, or in format incompatibilities. Better tool integration is needed.

Interpretation of meta-analysis results is not only done by the systematic reviewer. Clinicians and other users of the review may interpret the results and use their own interpretation.

### Systems approach to systematic review automation

A ‘living’ systematic review is updated at the time it is required to be used in practice [139]. It is no longer a document that summarizes the evidence in the past. Rather, it is designed and tested once, and is run at a push of a button to execute a series of search, appraisal, information extraction, summarization, and report generation algorithms on all the data available. Like other computer applications, the systematic review protocol itself can be modified, updated, and corrected and then redistributed so that such amendments are reflected in new systematic reviews based on the protocol.

Much research on integrated automatic review systems is still needed. Examples of complex systems that have evolved as combinations of simpler ones are abundant and provide hope as well as valuable components in a future system. In this survey, we found that systems designed to perform many of the systematic review tasks are already in wide use, in development, or in research. Semi-automated decision support systems will advance the end goal of completely autonomous systematic review systems.

An enterprise to automate systematic review has many stakeholders including clinicians, journal editors, registries, clinical scientists, reviewers, and informaticians. Collaboration between the stakeholders may include publication requirements of registration of reviews by medical editor committees, better adherence to CONSORT and PRISMA statements, and explicit PICO reporting. Accommodation in systematic review registries such as PROSPERO and standard data exchanges such as HL7 [140] may assist in the standardization and quality assurance of automatically generated systematic reviews.

The path to a fully automated systematic review system will continue to deliver a range of software applications that will benefit systematic reviewers directly as well as some that will benefit the community less directly. For example, imperfect applications that can extract data only from CONSORT-compliant abstracts can help editors conduct pre-submission quality assurance. Software that automatically differentiates citations to published papers from grey literature can also be used to create a new registry for grey literature. With small modifications, search strategies from previous systematic reviews can be used by guideline developers to find evidence summaries and identify gaps in knowledge.

## Conclusion

Synthesis for evidence based medicine is quickly becoming unfeasible because of the exponential growth in evidence production. Limited resources can be better utilized with computational assistance and automation to dramatically improve the process. Health informaticians are uniquely positioned to take the lead on the endeavor to transform evidence-based medicine through lessons learned from systems and software engineering. The limited resources dedicated to evidence synthesis can be better utilized with computational assistance and automation.

Conducting systematic reviews faster, with fewer resources, will produce more reviews to answer more clinical questions, keep them up to date, and require less training. Together, advances in the automation of systematic reviews will provide clinicians with more evidence-based answers and thus allow them to provide higher quality care.

## Abbreviations

AQE: automatic (search) query expansion; CI: confidence interval; CINAHL: Cumulative Index to Nursing and Allied Health Literature; CONSORT: consolidated standards of reporting trials; CRS: Cochrane Register of Studies; DARE: Database of Abstracts of Reviews of Effects; DOI: digital object identifier; HL7: Health Level 7; HR: hazard ratio; ISBN: International Standard Book Number; MeSH: Medical Subject Headings; ML: machine learning; NLG: natural language generation; NLP: natural language processing; NNT: number needed to treat; OCR: optical character recognition; PICO: Population (or Problem): Intervention: Control and Outcome; PRISMA: Preferred Reporting Items for Systematic Reviews and Meta-Analyses; PROSPERO: International Register of Prospective Systematic Review; RevMan: Review Manager; RR: relative risk; SD: standard deviation; TRIP: Turning Research Into Practice.

## Competing interests

The authors declare that they have no competing interests.

## Authors' contributions

GT designed and drafted the manuscript and conducted literature searches. PG provided domain guidance on systematic review tasks and processes. MKC conducted literature searches. AD provided domain expertise on clinical evidence and appraisal. FG conducted literature searches. EC provided domain guidance on decision support systems and informatics. All authors read and approved the final manuscript.
